# Effects of Heat-Killed *Lacticaseibacillus paracasei* MCC1849 on Immune Parameters in Healthy Adults—A Randomized, Double-Blind, Placebo-Controlled, Parallel-Group Study

**DOI:** 10.3390/nu16020216

**Published:** 2024-01-09

**Authors:** Kumiko Kato, Satoshi Arai, Soichiro Sato, Noriyuki Iwabuchi, Tsuyoshi Takara, Miyuki Tanaka

**Affiliations:** 1 Innovative Research Institute, R&D Division, Morinaga Milk Industry Co., Ltd., 5-1-83, Higashihara, Zama 252-8583, Japan; 2 Medical Corporation Seishinkai, Takara Clinic, 2-3-2-9, Higashigotanda, Shinagawa, Tokyo 141-0022, Japan

**Keywords:** *Lacticaseibacillus paracasei*, immune function, plasmacytoid dendritic cells, postbiotics, healthy adults

## Abstract

Previous clinical studies have shown that heat-killed *Lacticaseibacillus paracasei* MCC1849 suppresses subjective symptoms among healthy adults. However, the mechanism underlying this beneficial effect remains unclear. This clinical study aimed to investigate the effects of MCC1849 on immune functions in humans. In this randomized, double-blind, placebo-controlled, parallel-group study, 100 healthy adults were randomly divided into MCC1849 or placebo groups. Participants ingested test powder with 5 × 10^10^ MCC1849 cells or placebo powder for 4 weeks. Immune functions were evaluated using expression levels of CD86 and HLA-DR on dendritic cells (DCs), neutrophils, and natural killer cells. The expression levels of interferon (IFN)-α, -β, and -γ in peripheral blood mononuclear cells incubated with Cpg2216 in vitro were quantified. Efficacy analysis was performed on participants in the per-protocol set (placebo group; n = 47, MCC1849 group; n = 49). The expression level of CD86 on pDCs and the gene expression levels of IFN-α, -β, and -γ upon TLR9 agonist stimulation were significantly higher in the MCC1849 group at 4 weeks. No side effects were observed. This is the first report to show the positive effects of MCC1849 on human immune cells. These findings reveal one possible mechanism of how MCC1849 suppresses subjective symptoms.

## 1. Introduction

The common cold is a conventional term for a mild upper respiratory illness (URTI), which is the most frequent human disease [1,2]. The common cold is caused by a variety of viruses and bacteria, including coronaviruses, rhinoviruses, and adenoviruses. These viruses may be highly evolutionarily dynamic, and they have significant mutation rates, thus enabling them to evade preexisting immunity [3,4]. Because it is nearly impossible to respond to all sources of infection, it is important to enhance the immune response in the early stages of infection to prevent aggravation.

The host defense function mainly consists of two mechanisms: the innate immune system and the adaptive immune system. Every infectious microorganism possesses conserved molecular structures, such as lipopolysaccharide, peptidoglycan, flagellin, and microbial nucleic acids, which are collectively referred to as pathogen-associated molecular patterns (PAMPs) [5]. PAMPs are recognized by corresponding germline-encoded pattern recognition receptors (PRRs) expressed on innate immune cells of the host [6]. This triggers various signaling pathways to produce inflammatory responses and adaptive immunity [7,8]. Dendritic cells (DCs) are one of the key factors that express Toll-like receptors (TLRs) and form an essential interface between the innate sensing of pathogens and the activation of adaptive immunity. Naturally circulating DCs in humans can be classified into two main subsets based on phenotype and function: plasmacytoid DCs (pDCs) and myeloid DCs (mDCs) [9]. pDCs are useful for the detection and control of viral infections. pDCs express very high levels of TLR7 and TLR9 [10] and show a unique ability to produce large amounts of type I interferons (IFNs) upon sensing a virus [11]. Type I IFNs are also important for host defense because they contribute to signaling the infection and facilitating the communication among immune cells. Type I IFNs activate immune cells such as natural killer (NK) cells, B cells, and T cells [12,13]. It has been reported that 90% of URTIs are caused by viral infections [14]. Therefore, pDCs, including their production of type I IFNs, seemed to be particularly important to prevent infection and relieve the symptoms of URTIs.

Probiotics are live microorganisms that have beneficial physiological effects when administered in adequate amounts. Several meta-analyses suggest that probiotics could be beneficial in the context of the acute URTIs typically caused by viruses [15,16]. Probiotic bacteria possess conserved microbe-associated molecular patterns (MAMPs) and can activate TLRs, leading to the activation of nuclear factor-kappa B (NF-κB) and interferon regulatory factors (IRFs) in immune cells [15,17]. Postbiotics are defined as the preparations of inanimate microorganisms and/or their components that confer a health benefit on the host [18]. One of their health benefits is the modulation of the immune system [19]. Recently, the ingestions of some postbiotics have been reported to reduce cold-like symptoms in healthy adults by stimulating pDCs and showing positive effects on the production of type I IFNs [20,21,22,23].

Heat-killed *Lacticaseibacillus paracasei* MCC1849 (strain Shield, hereafter referred to as “MCC1849”) is also a type of postbiotic. Among lactobacilli, MCC1849 has been shown to have a high potential for interleukin-12 (IL-12) production [24]. IL-12 is a proinflammatory cytokine produced by DCs and phagocytes in response to pathogens and has been reported to contribute to host immune functions through, for example, inducing the production of IFN-γ [25]. The oral administration of MCC1849 increased IL-12 p40 gene expression in Peyer’s patches and antigen-specific IgA levels in the small intestine, lung, and serum in vivo. In addition, this in vivo study also demonstrated that the oral administration of MCC1849 prior to influenza virus infection resulted in significantly lower symptom scores and the significant inhibition of viral proliferation in the lung [24]. These data suggest the possibility that MCC1849 has beneficial effects on the immune response to pathogens. Two clinical studies showed that the long-term intake of MCC1849 suppressed subjective symptoms, such as the number of days and the duration of a stuffy nose and cold-like symptoms, in healthy adults [26,27]. In other words, it has been suggested that the ingestion of MCC1849 may help maintain the physical condition of healthy adults. However, these studies did not report the effects of MCC1849 on immune cells and cytokines. We hypothesized that the beneficial effects of MCC1849 on maintaining physical conditions may be due to the stimulation of pDCs and type I IFNs. Therefore, this randomized controlled study in healthy adults aimed to clarify the effects of MCC1849 on immune cells, including pDCs.

## 2. Materials and Methods

### 2.1. Study Design

This study was a randomized, double-blind, placebo-controlled trial conducted at Seishinkai Medical Association Inc., Takara Clinic (Tokyo, Japan) from September to December 2021. The study was performed in accordance with the Declaration of Helsinki (revised version of 2013) and the Ethical Guidelines for Medical and Health Research Involving Human Subjects. The study protocol was reviewed and approved by the institutional ethical review board at Seishinkai Medical Association Inc., Takara Clinic (reference number: 2109-00186-0038-1C-TC) on 15 September 2021. This study was registered at the University Hospital Medical Information Network Clinical Trial Registry on 21 September 2021 as UMIN000045531.

### 2.2. Participants

Japanese males and females were recruited and assessed for their eligibility to participate in the present study. The inclusion criterion was healthy adults aged 20–64 years at the time of informed consent. The exclusion criteria were as follows: (1) participants who were undergoing medical treatment or had a serious medical history of any disease, such as malignant tumour, respiratory disease, liver, kidney, heart, lung, digestive organ, blood, endocrine system, metabolic system, allergic disease, or autoimmune disease; (2) participants who were pregnant, breast-feeding, and planning to become pregnant during the experimental period; (3) participants who used drugs or ate functional foods that may affect immune function during the experimental period; (4) participants who participated in other clinical trials within the last 28 days before agreement; (5) participants who donated 400 mL or more of blood within 12 weeks before consumption of the test food or 200 mL or more of blood during the period of screening; (6) participants who planned to receive vaccination for infectious diseases; (7) participants who were deemed unsuitable for this study by the principal investigator for other reasons. Written informed consent was obtained from all participants.

### 2.3. Sample Size

The sample size was determined to be 50 participants per group based on previous similar reports [22,28] and the maximum number of participants that can be tested at the facility.

### 2.4. Randomization, Allocation, and Blinding

Participants were randomly assigned to two groups: the MCC1849 group and the placebo group, with an allocation rate of 1:1. Prior to the initiation of the present study, participants were randomly assigned according to simple randomization procedures (computerized random numbers) to 1 of 2 treatment groups by an independent allocation manager. The investigators, recruitment staff, and co-medical staff were blinded to the allocation list until the database and statistical plan were locked.

### 2.5. Intervention

Randomized participants received the test powder containing 5 × 10^10^ cells of MCC1849 and maltodextrin derived from tapioca starch as an excipient or matched placebo powder of maltodextrin without MCC1849 once daily for the 4 weeks of the intervention period. The test foods (test powder and placebo powder) were produced by Morinaga Milk Industry (Tokyo, Japan). Both powders were similar in appearance, weight (1 g), smell, taste, and packaging.

Participants were informed to prohibit them from taking medications and foods that may affect the immune system. The use of antibiotics, anti-allergic medications, special health foods, foods with functional claims, and dietary supplements containing *Lactobacillus*, *Bifidobacterium*, or oligosaccharides was prohibited during this study. If they intake these prohibited substances, they recorded it in the participant’s diary during the study period.

### 2.6. Outcomes

We evaluated the expression of activity markers (CD86 and HLA–DR) on DCs, phagocytic capacity, and the oxidative burst activity of neutrophils, natural killer (NK) cell activity, and the gene expression levels of IFNs. Blood samples were collected at baseline and after the ingestion period (4 weeks). The phagocytic capacity and oxidative burst activity of neutrophils and NK cell activity were investigated in LSI Medience (Japan) as previously described [29].

### 2.7. Preparation of Peripheral Blood Mononuclear Cells (PBMCs)

Peripheral blood was collected using a Venoject II VP-H100K with heparin sodium (Terumo Corporation, Tokyo, Japan). Immediately after collecting blood samples, peripheral blood mononuclear cells (PBMCs) were isolated by using Lymphoprep tubes (Serumwerk Bernburg AG, Bernburg, Germany) according to the manufacturer’s instructions. The isolated PBMCs were suspended in RPMI 1640 medium (10% FBS, 1% penicillin and streptomycin, cat #72400047; Thermo Fisher Science, Waltham, MA, USA) and stored at 4 °C until used for the analysis of DCs and cell culture.

### 2.8. FACS Analysis

PBMCs were stained with fluorescent dye conjugated to antibodies. For pDCs, BD Horizon Fixable Viability Stain 780 (FVS780) viability dye, PE-Cy7 Mouse Anti-Human CD123 (BD, 560826), BB515 Mouse Anti-Human Neuropilin-1 (CD304) (BD, 566036), APC Mouse Anti-Human CD86 (BD, 555660), and PE Mouse Anti-Human HLA-DR (BD, 556644) were used. For mDCs, FVS780, Anti-human Lineage Cocktail 1 (Lin1) (CD3, CD14, CD16, CD19, CD20, CD56) (BD, 340546), PE-Cy™7 Mouse Anti-Human CD11c (BD, 561356), APC Mouse Anti-Human CD86 (BD, 555660), and PE Mouse Anti-Human HLA-DR (BD, 556644) were used. After staining, both cell lines were fixed with BD Cytofix Fixation Buffer (BD Bioscience). CD123^+^CD304^+^ cells were defined as pDCs, and Lin1^−^ CD11c^+^ cells were identified as mDCs. The expression levels of HLA-DR and CD86 were used as activation markers of pDCs and mDCs. After staining, the cells were analyzed by flow cytometry using CytoFLEX (Beckman Coulter, Brea, CA, USA), and the data were analyzed using FlowJo 10.9.0 software (Treestar, ON, USA).

### 2.9. Cell Culture

PBMCs were adjusted to a final concentration of 1 × 10^6^ cells/mL with RPMI 1640 medium (10% FBS, 1% penicillin and streptomycin, cat#72400047; Thermo Fisher Science) and stimulated with or without 1 μg/mL TLR9 agonist ODN 2216 (InvivoGen, CA, USA) for 4 h at 37 °C and 5% CO_2_. Afterwards, the cells were harvested, mixed with Lysis Buffer LBP (MACHEREY-NAGEL GmbH & Co. KG, Duren, Germany), and stored at −80 °C until gene expression analysis.

### 2.10. RNA Extraction and Quantitative Real-Time PCR

Total RNA was isolated by NucleoSpin RNA Plus (MACHEREY-NAGEL GmbH & Co. KG, Duren, Germany). Complementary DNA (cDNA) was synthesized using PrimeScript RT Master Mix (Perfect Real Time) (Takara Bio Inc., Shiga, Japan). The cDNA was used in a real-time PCR containing a 0.1 mM concentration of both forward and reverse primers and TB Green^®^ Premix Ex Taq™ (Tli RNaseH Plus) (Takara Bio Inc., Shiga, Japan). The quantitation of fold induction was analyzed by the 2^−ΔΔCT^ method. β-Actin was used as the reference gene. The primers for β-actin, IFN-α, IFN-β, and IFN-γ were as previously described [21,30,31].

### 2.11. Safety Assessment

A safety assessment was performed on all participants who consumed the test foods at least once. All adverse events related to subjective and objective symptoms were recorded in the participant’s diary during the study period. The severity of the adverse events and association with the test foods were evaluated by the principal investigator. The number and incidence of adverse events were calculated for each group.

### 2.12. Statistical Analysis

The efficacy analysis was performed on the per-protocol set (PPS). For between-group comparisons of the geometric mean fluorescence intensity (MFI) of HLA-DR and CD86, the phagocytic and oxidative burst activity of neutrophils and NK cell activity at 4 weeks was performed by using analysis of covariance (ANCOVA) with adjustments for the values at baseline. Within-group comparisons were performed by using paired *t*-tests. Between-group comparisons of the gene expression of IFNs after culture were performed by using the Mann–Whitney U test. In the case of any missing values, the values were not complemented. The baseline demographics were compared by Fisher’s exact test or Welch’s *t*-test. Statistical analysis was performed using SPSS software version 26 (IBM, Tokyo, Japan), with significance set at a *p* value < 0.05.

## 3. Results

### 3.1. Study Flow and Baseline Characteristics of Participants

**Figure 1:**
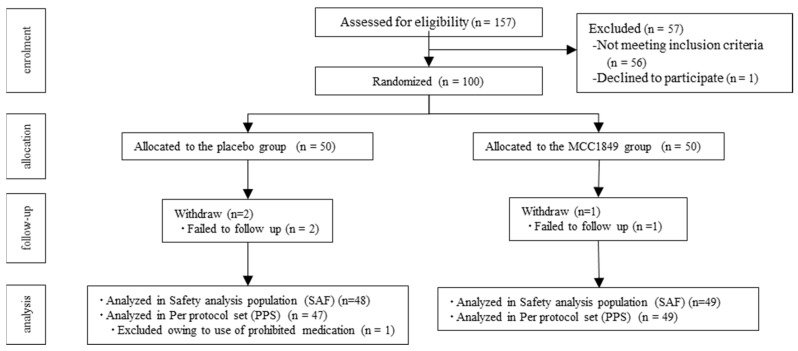
Flow diagram of the study.

Participants were recruited from September to October 2021. Figure 1 shows the participant flow diagram. Consent was obtained from 157 of the participants, and enrolment was completed for a total of 100 participants who were identified by the principal investigator as eligible healthy adults. The participants were then randomized to two groups (placebo group, n = 50; MCC1849 group, n = 50). Three participants were lost to follow-up, and their post-randomization data were not available. The remaining 97 participants were analyzed in the safety analysis population. One participant in the placebo group was excluded from the efficacy analysis owing to the use of prohibited medication. There was no intake of prohibited foods or supplements during the study period. As a result, 47 participants from the placebo group and 49 participants from the MCC1849 group were included in the efficacy analysis as the PPS. The characteristics of the study participants at baseline are shown in Table 1. There was no significant difference between the two groups in the characteristics of the study participants at baseline. The ingestion rates of the test foods were 100% for all participants in both groups.

### 3.2. Effects on the Activation Markers on pDCs and mDCs

The geometric mean CD86 and HLA-DR expression levels on DCs are shown in Table 2. The expression level of CD86 on pDCs was significantly higher in the MCC1849 group than in the placebo group at 4 weeks. The other parameters, the expression levels of CD86 on mDCs and HLA-DR on DCs, did not significantly differ between the two groups.

### 3.3. Effects on the Activity of Neutrophils and NK Cells

No significant differences were observed in the phagocytic capacity and oxidative burst activity of neutrophils and the cytotoxic activity of NK cells among the groups (Table 3).

### 3.4. Effects on the Immune Response of PBMCs against Cpg2216

The gene expression levels of IFN-related genes in PBMCs are shown in Table 4. Due to the limited number of PBMCs in several blood samples, there were some missing values. At 4 weeks, the expression levels of IFN-α, IFN-β, and IFN-γ in the MCC1849 group were significantly higher than those in the placebo group. In both groups, the expression levels of IFN-α and IFN-β were significantly lower at 4 weeks than at baseline. In the placebo group, the expression levels of IFN-γ at 4 weeks tended to be lower than those at baseline.

### 3.5. Safety Assessment

A total of 13 participants reported adverse events during the study. All adverse events were mild and judged to be unrelated to the dietary intervention. No significant differences were noted in the incidence of adverse events among the groups (9/48 participants in the placebo group and 4/49 participants in the MCC1849 group; *p* value = 0.147).

## 4. Discussion

This is the first study to investigate the effects of the ingestion of MCC1849 on immune cells in healthy adults. We have previously reported that MCC1849 may have the potential to help maintain the physical condition of healthy adults [26,27]. In addition, MCC1849 enhances antigen-specific IgA production and likely affects T-cell differentiation in Peyer’s patches in vivo. However, the interaction between MCC1849 and host immune cells in humans is still unclear. In the present study, our results showed that the ingestion of MCC1849 for 4 weeks increased pDC activity in the peripheral blood and maintained the transcription of both type I and type II IFNs stimulated by the TLR9 ligand.

In this study, pDC activity in PBMCs was assessed using the expression levels of CD86 and HLA-DR. The ingestion of MCC1849 for 4 weeks significantly increased the expression of CD86, while the expression of HLA-DR was not increased (Table 2). CD86 is a co-stimulatory ligand for CD28 on T cells, and its interaction is important for T cell activation and cytokine production [32]. HLA-DR is an MHC class II molecule and presents exogenous antigens [33]. Although both markers are important for the immune system, the behavior of all pDC activity markers was inconsistent even in lactobacilli, which have been reported to maintain immune functions [34]. Interactions between host intestinal cells and probiotics are thought to mainly occur at the surface of the intestinal barrier, including the intestinal epithelium and the underlying lamina propria. Probiotics are processed directly by DCs in the lamina propria in the intestinal lumen and/or transferred to DCs via specialized enterocytes known as microfold cells (M cells) located in the epithelium overlying Peyer’s patch [35]. Some heat-killed lactobacilli, such as *L. lactis* JCM 5805 and *L. acidophilus* L-92, have been suggested to be taken up by Peyer’s patches, including M cells, interact with pDCs, and enhance the expression of CD86 on human pDCs [36,37]. Our in vivo study previously reported that the oral administration of MCC1849 enhances antigen-specific IgA production and likely affects Tfh cell differentiation in Peyer’s patches [24]. Therefore, we consider that the enhanced CD86 expression in this study is caused by the contact between MCC1849 and pDCs in the intestine.

We evaluated IFN responses to the TLR9 agonist CpG2216 simulating infection conditions, since activated pDCs are expected to stimulate the production of type I IFNs. The immunological response test using PBMCs cultured with CpG2216 showed that MCC1849 would maintain the transcriptional levels of IFN-α, -β, and -γ (Table 4). Contrary to our expectation, the transcriptional levels of IFN-α and -β were lower at 4 weeks than at baseline in both the placebo and the MCC1849 groups. The influence of environmental factors, such as changes in temperature, was considered, but it was difficult to determine the factors from the data obtained in this study. Even in this situation, the ingestion of MCC1849 showed the effects of alleviating the decrease in the gene expression of type I IFNs. IFN-mediated antiviral responses are central to host defense against viral infections. Human pDCs mainly express the IFN-α and IFN-β subtypes, which act in an autocrine and paracrine manner to initiate cellular and intercellular processes to prevent the spread of viruses and promote the elimination of virus-infected cells. pDCs are responsible for 95% of type I IFNs production by mononuclear cells, as they are able to produce 200–1000 times more type I IFNs than any other white blood cell after microbial exposure [12,38]. Mainly through type I IFNs production, pDCs provide an initial line of host defense against viral infection [38,39,40]. Type I IFNs support other immune cell functions, such as NK cells, and induce the maturation and activation of DCs. The support affects both innate immunity and acquired immunity. Type I IFNs support the activation and expansion of antigen-specific CD4+ helper T (Th) cells and CD8+ cytotoxic T cells and contribute to the differentiation of follicular Th cells (Tfh cells), which are critical for the induction of B-cell responses [38,41]. Because pDCs affect the entire immune system, the activation of pDCs seemed to contribute to maintaining the production of the type I IFN response to infections and alleviating subjective symptoms in healthy adults. Some clinical reports have shown that the ingestion of postbiotics has increased or maintained the expression level of CD86 on pDCs and the production of IFN-α, suppressing the onset of cold-like symptoms [20,22,23,28,42,43,44]. For example, one of these reports showed that the daily ingestion of heat-killed *L. lactice* JCM5805 reduced the number of incidence days of cold-like symptoms such as “Sneeze or running nose” and “Cough” in healthy adults. At the same time, they also reported that the ingestion contributed to the activation of pDCs and the higher expression of IFN-α in PBMCs. These results have indicated that the relationships of pDC-related anti-viral immunity suppress the incidence of cold-like symptoms [44]. These reports support our proposed mechanism of MCC1849 in protection against infection. In other words, the ingestion of MCC1849 seemed to contribute to activating pDCs, maintaining the induction of type I IFNs against infection and reducing cold-like symptoms.

The maintenance of the transcriptional level of IFN-γ was also demonstrated in this study. IFN-γ is a potent activator of the antimicrobial functions of phagocytes and contributes to appropriate immune responses. IFN-γ is a type II IFN and is produced predominantly by NK cells and activated T cells [45]. We consider that the maintenance of the transcription level by MCC1849 may involve immune factors other than pDCs. Type I IFNs, IL-12, IL-18, and IL-15 are potent activators of the NK cell effector function [46]. We previously reported that MCC1849 induced the highest levels of IL-12 when compared with the effects of other Lactobacillus type strains [24]. We speculate that type I IFNs and IL-12 contribute to maintaining the expression level of IFN-γ by MCC1849. Based on the results of the maintenance of IFNs, it is possible that MCC1849 has a broad effect on immune cells in humans.

There was no significant difference in the activation markers of mDCs, NK cell activities, or neutrophil activities. Generally, the evaluation of the effects of probiotics on these immune cells is needed to select suitable participants and evaluate them by other methods [47].

There were no side effects associated with the ingestion of test food for 4 weeks. A previous study similarly demonstrated the safety of MCC1849 for 24 weeks [27]. MCC1849 has also obtained the self-affirmed “generally recognized as safe” (GRAS) notification for the intended use of conventional foods. MCC1849 has been verified to be safe for a long time.

There were some limitations in this study. This study focused on the effects on immune cells for 4 weeks. It was difficult to assess the physical condition of the patients because of the limited number of participants and short study period. Further studies evaluating the effects on subjective symptoms and immune cells in the same study may elucidate the detailed effects of MCC1849 on immune function.

## 5. Conclusion

The ingestion of 5 × 10^10^ cells of MCC1849 by healthy adults activates pDCs and maintains the expression levels of IFNs, IFN-α, β, and γ under conditions that mimic infection. This is the first report to show the beneficial effects of MCC1849 on immune cells in humans. Because pDCs have a crucial role in maintaining healthy conditions, we consider that the activation of pDCs by the ingestion of MCC1849 contributes to appropriate immune responses and may have the potential to suppress subjective symptoms in healthy adults.

## Figures and Tables

**Table 1:** Characteristics of the study participants at baseline (PPS).

Parameter	Placebo Group	MCC1849 Group	*p* Value
Number (Male/Female)	47 (14/33)	49 (14/35)	1.000
Age (y)	43.4 ± 11.0	44.3 ± 10.6	0.669
Height (cm)	163.2 ± 8.3	163.8 ± 8.6	0.744
Weight (kg)	58.3 ± 10.2	60.0 ± 11.2	0.431
BMI (kg/m^2^)	21.8 ± 2.8	22.2 ± 2.8	0.453

Values are mean ± SD. *p* value, estimated by Fisher’s exact test or Welch’s *t*-test.

**Table 2:** CD86 and HLA-DR expression levels on DCs.

				Baseline	4 Weeks	
		Group	n	Mean (SE)	Mean (SE)	*p* Value
pDCs	CD86 MFI	Placebo	47	1732.0 (74.7)	1665.9 (48.4)	0.037
		MCC1849	48	1674.5 (51.2)	1764.6 (53.4)	
pDCs	HLA-DR MFI	Placebo	47	76,190.9 (3667.8)	80,225.3 (3925.4) *	0.933
		MCC1849	48	78,657.8 (4191.1)	82,813.9 (4427.6) *	
mDCs	CD86 MFI	Placebo	47	8512.8 (289.9)	9376.2 (184.3) *	0.243
		MCC1849	48	8505.0 (289.6)	9094.3 (192.2) *	
mDCs	HLA-DR MFI	Placebo	47	150,305.9 (6179.8)	143,595.4 (7363.0)	0.358
		MCC1849	48	145,741.5 (5594.2)	145,990.5 (6663.1)	

*p* value, estimated by analysis of covariance (ANCOVA) adjusted for baseline values. * *p* value < 0.05, comparison between baseline and 4 weeks by paired *t*-test.

**Table 3:** Effects on the activity of neutrophils and NK cells.

			Baseline	4 Weeks	
		n	Mean (SE)	Mean (SE)	*p* Value
Neutrophil phagocytic capacity (%)	Placebo	47	84.6 (1.2)	84.3 (1.6)	0.987
	MCC1849	49	84.5 (1.5)	84.2 (1.9)	
Neutrophil oxidative burst activity (%)	Placebo	47	95.1 (1.2)	95.1 (1.4)	0.986
	MCC1849	49	94.9 (1.2)	95.0 (1.2)	
NK cell activity (%)	Placebo	47	58.2 (15.8)	58.8 (18.2)	0.317
	MCC1849	49	60.3 (17.7)	58.4 (19.6)	

*p* value, estimated by analysis of covariance (ANCOVA) adjusted for baseline values.

**Table 4:** Gene expression levels of IFN-related genes in PBMCs stimulated by CpG2216.

		Baseline		4 Weeks		
		n	Median (IQR)	n	Median (IQR)	*p* Value ^#2^
IFN-α	Placebo	44	49.6 (14.5–105.7)	47	13.8 (6.1–27.2)	<0.001
	MCC1849	47	42.7 (17.6–178.0)	48	21.7 (11.8–38.3)	<0.001
	*p* value ^#1^		0.237		0.026	
IFN-β	Placebo	44	66.3 (33.5–140.9)	47	36.2 (14.7–57.2)	<0.001
	MCC1849	47	64.3 (30.6–237.2)	48	49.7 (27.8–82.3)	<0.001
	*p* value ^#1^		0.368		0.045	
IFN-γ	Placebo	44	3.4 (2.2–6.7)	47	2.7 (1.7–5.4)	0.062
	MCC1849	47	4.2 (2.3–7.4)	48	4.1 (2.5–6.7)	0.863
	*p* value ^#1^		0.330		0.017	

*p* value ^#1^, comparison between groups by the Mann–Whitney U test. *p* value ^#2^, comparison between baseline and 4 weeks by the Wilcoxon signed-rank sum test.

## Data Availability

The data presented in this study can be found in this published article.
